# Experimental Study on Noise-Reduced Propagation Characteristics of the Parametric Acoustic Array Field in a Neck Phantom

**DOI:** 10.3390/s25030802

**Published:** 2025-01-29

**Authors:** Li Wang, Fengji Li, Jie Zhou, Haijun Niu

**Affiliations:** School of Biological Science and Medical Engineering, Beihang University, Beijing 100191, China

**Keywords:** electrolarynx, parametric acoustic array, radiation noise, tissue mimicking phantom

## Abstract

The electrolarynx (EL) is a common device for voice reconstruction in laryngectomy patients, but its mechanical sound source generates significant radiation noise, affecting the naturalness and acceptability of the speech. The parametric acoustic array (PAA), which produces directionally propagated difference-frequency sound waves, presents a promising alternative for creating a more natural glottal-like voice source in the trachea while reducing radiation noise. In this study, we developed a tissue-mimicking phantom to simulate human neck tissue and used a single-transducer-based PAA platform to generate modulated ultrasound signals with glottal waveform characteristics. Ultrasonic microphones captured sound signals fromthe trachea and surrounding air, and signal processing was used to isolate the difference-frequency signals. The results demonstrated that difference-frequency signals were successfully detected in the phantom’s trachea, with time-domain waveforms and frequency spectra closely resembling the designed glottal waveform (Pearson correlation coefficient = 0.9438). Additionally, radiation noise produced by the PAA was significantly lower (23 dB, *p* < 0.0001) compared to the traditional EL. These findings demonstrate the potential of PAA for voice source reconstruction in laryngectomy patients and suggest its capacity to enhance speech rehabilitation outcomes. Further research is required to refine the frequency range and evaluate clinical applicability.

## 1. Introduction

The electrolarynx (EL) is a critical device for voice reconstruction in patients undergoing total laryngectomy, a surgical procedure that removes the larynx [1]. This device compensates for the loss of vocal cords by using an external sound source, offering benefits like ease of use and the ability to produce continuous speech [2]. Research shows that more than half of laryngectomy patients rely on EL as their primary means of communication within five years post-surgery [3].

The EL functions by placing a vibrating membrane against the neck, allowing the sound produced to travel through surrounding tissues to the pharynx, thereby creating a substitute voice. However, a significant drawback is the mechanical noise (often referred to as “radiation noise”) generated by the device, which can reach 20–25 dB and severely interfere with speech intelligibility [4,5].

Researchers have employed various strategies to eliminate radiation noise, including physical modifications to the EL and signal processing techniques. For instance, Madden et al. [6] replaced the traditional motor with an eccentric motor, achieving a 20% improvement in speech intelligibility by reducing noise. However, maintaining clear and natural speech quality remains a challenge. Norton and Bernstein [7] wrapped the EL in thick foam, which reduced radiation noise by approximately 5 dB. However, this modification made the device cumbersome and less user-friendly. Other studies have focused on signal processing methods, such as spectral subtraction, voice conversion, and adaptive filtering, which have shown promise in enhancing speech quality by minimizing background noise [8,9,10,11]. Although these techniques enhance the clarity of the output, their lack of real-time performance often makes them unsuitable for spontaneous conversation.

The primary source of radiation noise in electrolarynx (EL) devices originates from external sound sources. One potential solution is to relocate the sound source to the oral cavity or pharynx, a method that has been explored by several researchers. For instance, Takahashi et al. [12,13] mounted a vibrating source on a denture; however, the resulting sound failed to produce natural voice quality. Huang et al. [14] developed a sound-generating device that is placed on the upper jaw, using a 3D-printed speaker holder to secure the speaker to a tooth sleeve. This design allows users to modulate sound frequency and amplitude by adjusting lung pressure and mouth shape. Despite its advantages, the tooth sleeve can interfere with speech production and pose challenges related to stability and hygiene. Moreover, foreign objects in the mouth can further complicate articulation. Painter et al. [15] explored an electromagnetic EL device for implantation in neck tissue, but this approach carries the risks associated with surgical procedures.

Parametric acoustic array (PAA) technology offers a promising alternative to address these challenges. This technique, which has been successfully applied in fields such as underwater measurement [16,17], underwater communication [17], and parametric speakers [18,19,20], generates difference-frequency waves from high-frequency sound waves. This capability enables precise and focused sound generation, even in complex environments [21]. Mills et al. [22] demonstrated the feasibility of generating difference frequency signals in soft tissue, highlighting the potential of using PAA for internal voice source reconstruction. While simulations have provided valuable insights, they cannot fully replicate the complexity of real tissue environments. Experimental validation is therefore essential to confirm the feasibility of using PAA in practical applications. To bridge this gap, our study conducts experimental investigations using a tissue-mimicking phantom to explore the feasibility of generating voice sources within the human body through PAA technology.

This study aims to experimentally investigate the use of modulated PAA technology to generate voice sources within a tissue-mimicking phantom that replicates the acoustic characteristics of human neck tissue. By comparing generated voice sources with natural voice and traditional EL outputs, this study seeks to establish the effectiveness of PAA technology in voice reconstruction. The findings aim to provide critical insights into the practical application of PAA technology for voice reconstruction in laryngectomy patients, with the potential to significantly improve their quality of life.

## 2. Materials and Methods

### 2.1. Experimental Platform

**Figure 1:**
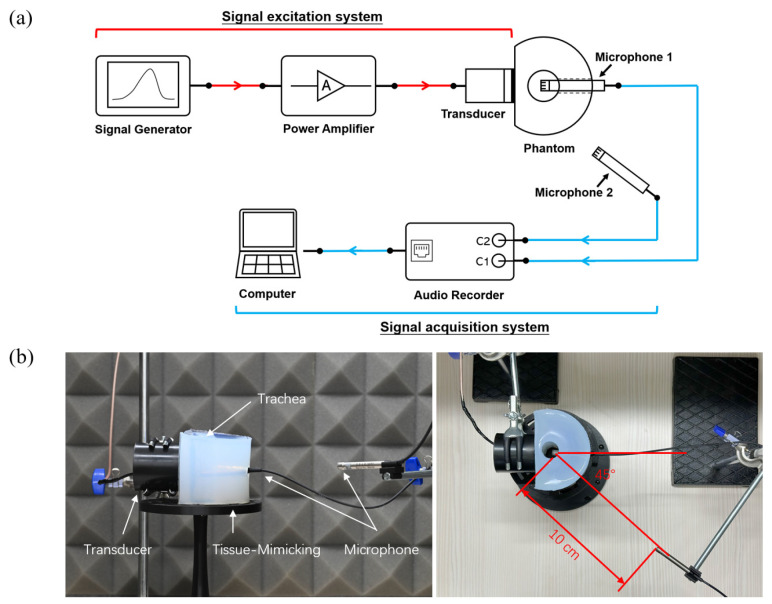
Experimental setup for investigating the propagation characteristics of the PAA difference-frequency sound field in a neck phantom. (**a**) Schematic diagram of the experimental platform; (**b**) photograph of the physical setup of the system: the ultrasound transducer is placed on the surface of the phantom, with coupling gel applied in the center. A microphone (Microphone 1) is inserted into a hole on the opposite side of the phantom, with its tip positioned at the center of the phantom’s trachea to capture the sound field inside the trachea. Another microphone (Microphone 2) is placed 10 cm from the trachea center on the outside of the phantom, at the same horizontal plane as Microphone 1 and at an approximate 45° angle to Microphone 1, to collect radiated noise.

The experimental platform was designed to investigate the propagation characteristics of the PAA difference-frequency sound field in a neck phantom. The setup comprises two main subsystems: the signal excitation system and the signal acquisition system, with a neck phantom serving as the medium for acoustic wave propagation. The complete experimental setup is illustrated in Figure 1, which includes a schematic diagram (Figure 1a) and a photograph of the physical setup (Figure 1b).

#### 2.1.1. Human Neck Tissue-Mimicking Phantom

In this study, a polyvinyl alcohol (PVA) material (product No. 563900, Sigma-Aldrich) was used to fabricate an acoustic phantom that simulates human neck tissue. The PVA (molecular weight: 130,000, Sigma-Aldrich, Zwijndrecht, The Netherlands) was prepared by dissolving 20% wt PVA in a mixture of 80% wt dimethyl sulfoxide (DMSO, Sigma-Aldrich, Zwijndrecht, The Netherlands) and 20% wt Milli-Q water. The solution underwent a series of freezing and thawing cycles to achieve the desired acoustic properties, which were designed to approximate those of human tissue (speed of sound: 1616 m/s, attenuation coefficient: 1.69 dB/cm, B/A value: 11.7) [23]. The phantom was shaped as a hollow cylinder, with a flat surface on the front for precise placement of the ultrasonic transducer. The height of the phantom was approximately 10 cm, and the outer diameter of the cylinder was designed to match the diameter of a human neck. The diameter of the central hole was chosen to resemble the size of the lower human vocal tract. Additionally, the thickness from the inner hole to the flat surface was made to approximate the thickness of human neck tissue [24]. A circular hole, approximately 1 cm in diameter, was added to the rear of the phantom to allow the insertion of a microphone for measurement.

#### 2.1.2. Signal Excitation System

The signal excitation system was designed to generate stable ultrasonic signals. The excitation signal was produced by an arbitrary waveform generator (Analog Discovery 3, Digilent, WA, USA) and then amplified by a power amplifier (ATA3040, Aigtek, Xi’an, China) to drive the ultrasonic transducer (H2KA050 KA1CD00, Unictron, Taiwan). The transducer, with a center frequency of 50 kHz and a diameter of 5 cm, was positioned at the middle of the flat surface of the phantom, ensuring optimal transmission of acoustic waves into the medium. Figure 1b illustrates the physical setup of the transducer and phantom.

### 2.2. Experimental Procedure

The experiments were conducted in a quiet room to minimize external noise interference. The signal excitation system transmitted an excitation signal with a peak-to-peak voltage of 90 V, driving the ultrasonic transducer to emit ultrasound waves, which propagated through the neck phantom. The signal acquisition system captured 10 s of continuous audio data at a sampling rate of 204.8 kHz with 24-bit resolution. To assess the impact of the phantom’s acoustic properties, measurements were also taken in air after the phantom was removed. All measurements were repeated three times for reliability.

Subsequently, the ultrasonic transducer was replaced with a commercial EL, and the same acquisition conditions were used to capture the sound emitted by the EL as it propagated through the phantom.

### 2.3. Excitation Signal

**Figure 2:**
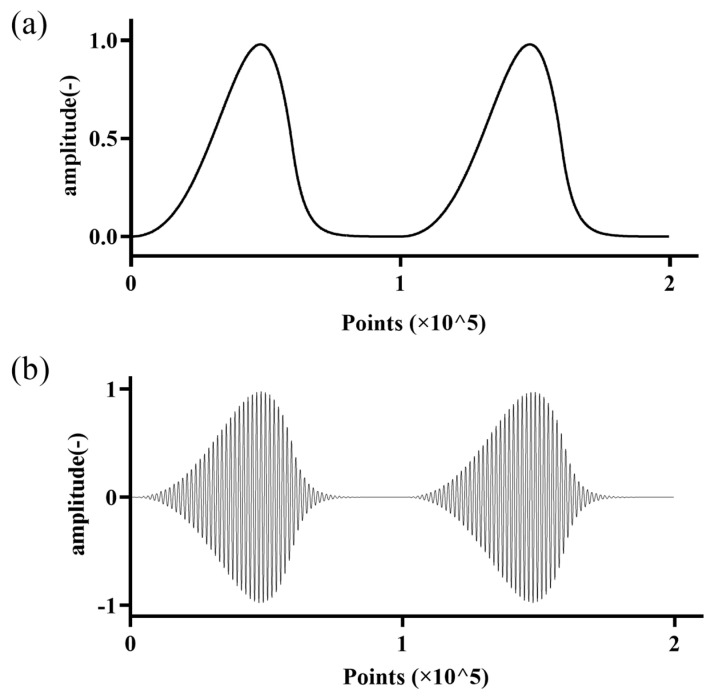
(**a**) Predefined glottal waveform and (**b**) corresponding AM-modulated excitation signal.

The parametric array excitation signal used in this study was generated using the Amplitude Modulation (AM) method [25], as described by the following equation:

(1) $$S\left(t\right)=(A+m(t))\times cos(2\pi f_{c}t),$$

where m(t) represents the envelope signal, s(t) is the resulting modulated signal, A is the amplitude of the carrier signal, and $cos(2\pi f_{c}t)$ is the carrier signal with frequency $f_{c}$. The envelope signal was generated using the Liljencrants–Fant (LF) glottal waveform model, which accurately simulates human glottal airflow during phonation [26]. The glottal waveform frequency was set to 200 Hz, with a carrier frequency of 50 kHz corresponding to the central frequency of the ultrasonic transducer. The modulation depth was set to 100%, resulting in full modulation of the carrier signal by the glottal waveform. Figure 2 illustrates the predefined glottal waveform signal and the corresponding AM-modulated excitation signal.

### 2.4. Signal Processing and Parameter Evaluation

The collected sound signals were subjected to a finite impulse response (FIR) band-pass filter with a passband of 20 Hz to 1000 Hz to extract the difference frequency signals. The extracted signal waveform was compared to the preset glottal waveform in the time domain. To quantify the similarity between the two signals, the Pearson correlation coefficient (r) was calculated. This was performed using MATLAB, with r computed based on the covariance of the variables normalized by their standard deviations:

(2) $$r=\frac{\sum \left(x_{i}-\bar{x}\right)\left(y_{i}-\bar{y}\right)}{\sqrt{\sum {\left(x_{i}-\bar{x}\right)}^{2}\sum {\left(y_{i}-\bar{y}\right)}^{2}}}$$

where $x_{i}$ and $y_{i}$ represent the measured values of AS/TMS and LFS, respectively, and $\bar{x}$ and $\bar{y}$ are the mean values of AS/TMS and LFS.

Additionally, the autoregressive (AR) power spectral density of the acquired signals was calculated using the AR Burg method, with an order of 190 chosen for its stability and accuracy in spectral estimation.

The sound pressure levels (SPLs) recorded by the external and internal microphones were used to evaluate the intensity of the difference frequency signal and quantify radiation noise. The SPL difference (Δ*L*) between the external microphone, which measures radiation noise, and the internal microphone, which measures the sound inside the phantom, serves as an indicator of noise leakage. A larger Δ*L* indicates less radiation noise. The SPL difference was calculated using the following formula:

(3) $$∆L={SPL}_{ext}-{SPL}_{int},$$

To assess the statistical significance of the SPL differences between the two excitation sources (EL and PAA), a paired *t*-test was performed using a significance level of p<0.001. This stricter threshold was chosen to ensure robust conclusions in the context of this study.

## 3. Results

### 3.1. Time-Domain Analysis

**Figure 3:**
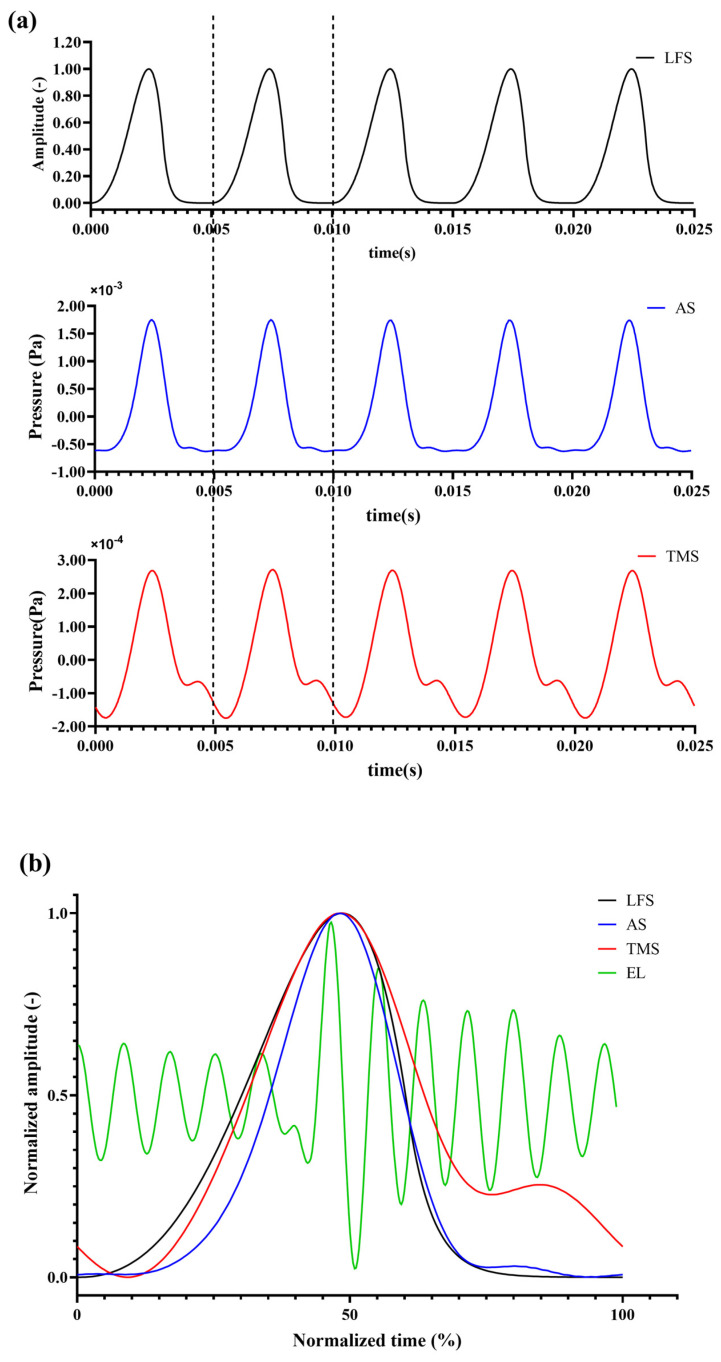
(**a**) Time−domain waveforms of LFS, AS, and TMS. (**b**) Normalized comparison of one period from the LFS, AS, TMS, and EL signals, derived from one cycle in Figure 3a (between the two dotted lines), highlighting waveform distortions and differences in peak and rise times. In contrast, the excitation signal produced by the EL is completely different from the glottal wave signal. The Pearson correlation between the EL signal and the LFS is only 0.0057, indicating minimal similarity.

The waveforms of the difference-frequency glottal wave obtained after excitation by the parametric array are shown in Figure 3. Figure 3a illustrates the waveform for five periods, with the first row representing the glottal wave obtained through the LF model calculation (envelope signal, LFS). The second and third rows show the difference-frequency signal waveforms captured at the transducer’s axial position in air (air signal, AS) and after propagation through the tissue-mimicking phantom (tissue-mimicking signal, TMS), respectively.

From the time-domain signals, it can be observed that the modulated glottal wave signal, after propagating through both the air and tissue-mimicking media, retains a periodicity corresponding to the fundamental frequency (F0) of the original glottal wave signal. The restored waveforms closely resemble the original glottal wave signal. Pearson correlation analysis revealed a correlation coefficient of 0.9767 between AS and LFS, and 0.9438 between TMS and LFS.

Compared to the LFS waveform, the difference-frequency waveforms (AS and TMS) show some distortion in their waveform shapes. To further analyze the differences, one period from the LFS, AS, and TMS waveforms, as well as the corresponding EL signal, were normalized and aligned by their signal peaks, as shown in Figure 3b. The analysis indicates that the peak of all three waveforms occurs at approximately the 50% point of the signal period. However, compared to LFS, the rise time of AS and TMS is steeper. The trough of the LFS signal is stable, without high-frequency noise or unwanted frequency components. On the right side of the peak, at approximately 70% of the signal period, a second sharp peak appears in both AS and TMS signals, with the TMS signal showing a more pronounced peak.

### 3.2. Frequency-Domain Analysis

**Figure 4:**
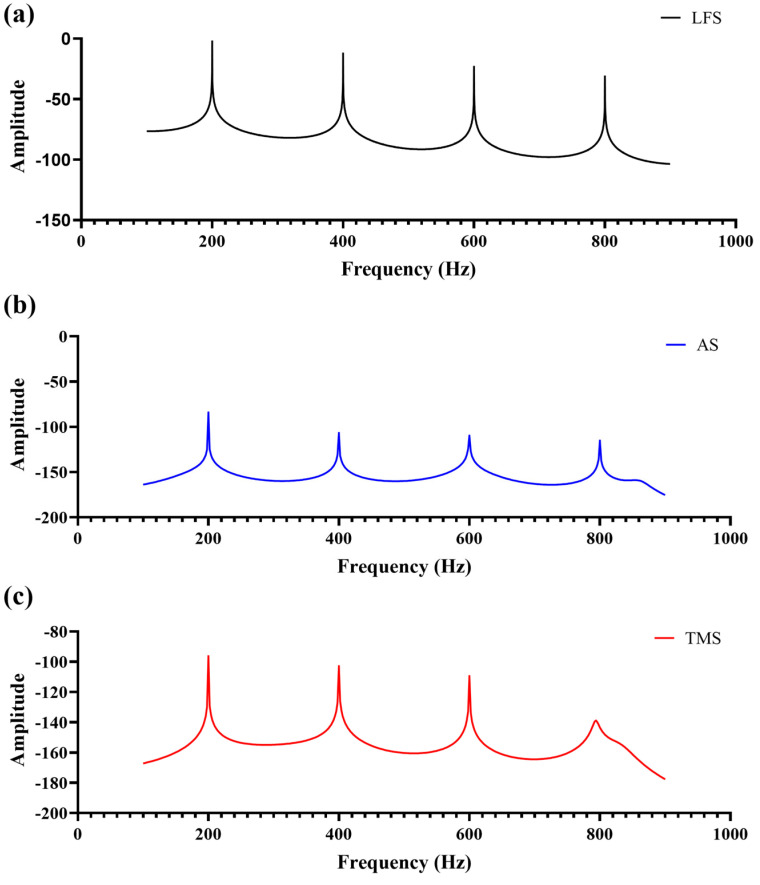
Burg AR power spectral density (PSD) curves of (**a**) LFS, (**b**) AS, and (**c**) TMS signals.

The Burg AR power spectral density (PSD) curves of LFS, AS, and TMS signals are shown in Figure 4. All three signals exhibit a dominant peak at the F0, with harmonic components appearing at integer multiples of the F0 (200 Hz, 400 Hz, 600 Hz, etc.). The energy of the F0 is the highest, while the harmonic components decrease in amplitude as the frequency increases.

The primary energy is concentrated around 200 Hz, with harmonic components becoming progressively weaker at higher frequencies. The fourth harmonic in the TMS signal is notably smaller compared to the AS signal. The frequency spectra of AS and TMS signals show slight attenuation of higher harmonics compared to the LFS signal, with the TMS signal exhibiting more pronounced attenuation in the higher frequency range.

### 3.3. Radiation Noise Analysis

**Figure 5:**
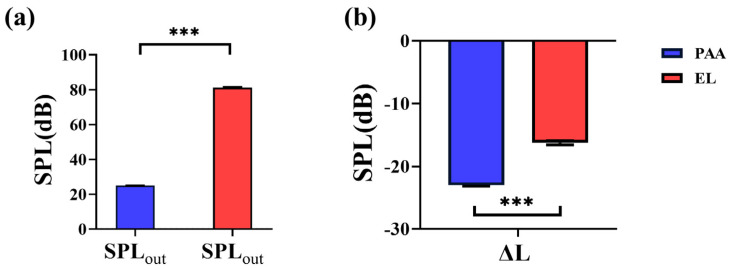
Comparison of radiation noise levels: (**a**) SPL of radiation noise for EL and PAA excitation sources, and (**b**) SPL difference (Δ*L*) between external and internal microphones for EL and PAA (***: *p* < 0.001).

As shown in Figure 5a, the sound pressure level (SPL) of radiation noise measured with the EL as the excitation source was 81.20 dB (SD: 0.36 dB). In contrast, when using the PAA as the excitation source, the SPL of radiation noise was significantly lower at 24 dB (SD: 0.16 dB), with a difference that was statistically significant (*p* < 0.0001).

Figure 5b illustrates the Δ*L* between the external and internal microphones. For the EL, the SPL difference was −16 dB (SD: 0.34 dB), whereas for the PAA, the SPL difference was −23 dB (SD: 0.15 dB). The SPL difference for the PAA was significantly higher than that for the EL (*p* < 0.0001), indicating that less radiation noise was leaking outside the phantom when using the PAA.

## 4. Discussion

This study experimentally investigates the propagation of the PAA sound field through tissue-mimicking media to evaluate its potential for reconstructing glottal waveforms. The results confirm that the PAA effectively generates low-frequency difference waves within the tissue-mimicking media, retaining the envelope characteristics of the modulated signal after propagation. Notably, these difference-frequency waves, when transmitted through the medium, exhibit a higher degree of similarity to the human glottal waveform than the excitation signal generated by the EL. This finding demonstrates the feasibility of using the PAA to reconstruct glottal waveforms, offering a potential advantage over traditional EL devices, which produce signals that are significantly different from natural human phonation.

The time–domain analysis revealed that the PAA emitted modulated glottal waveforms (LF model) maintaining periodicity with the same fundamental frequency (200 Hz) as the pre-defined signal, regardless of whether they propagated through air or tissue-mimicking media. The waveforms produced by PAA showed a high degree of similarity to the original glottal waveform, as evidenced by the Pearson correlation coefficients exceeding 0.9. In contrast, the waveform produced by the EL resembled a sharp, impulsive signal, rather than a smoothly modulated glottal waveform. This finding aligns with the simulations reported by Mills et al. [22] but differs in that Mills’ approach used a frequency difference method with two transducers, which could only generate sine waves corresponding to the frequency difference between two excitation signals. This study, however, utilized a single transducer to modulate the excitation signal, allowing for more versatile waveform generation and yielding a signal closer to natural human phonation. Furthermore, the use of one transducer simplifies the design of future devices, making the system more portable and cost-effective.

When comparing the LFS, AS, and TMS waveforms, the peak-to-peak value of the TMS waveform was significantly smaller than that of the AS waveform, possibly due to stronger attenuation of the sound by the tissue-mimicking medium. Additionally, the rise and fall of the TMS waveform were less smooth compared to the LFS waveform, with added peaks and valleys, which could be attributed to the harmonic components present in the original glottal signal. These harmonic components experienced some loss during propagation through the tissue-mimicking medium, a phenomenon that became more pronounced as the medium’s nonlinearity increased. This was further corroborated by the power spectral analysis.

In the frequency domain, the AR power spectral density analysis indicated that, in addition to the F0, the LF model waveform contained several harmonic components. After propagation through both air and tissue-mimicking media, the parametric array sound field retained these harmonic components, with their energy gradually decreasing as the frequency increased, similar to the energy distribution observed in the original LF model. When compared to the EL-generated sound field, the PAA signal exhibited a frequency spectrum that more closely matched that of the LF model, emphasizing its potential to more accurately simulate human phonation.

A key aspect of this study was the evaluation of radiation noise produced by the two excitation sources, using an external microphone placed behind the tissue-mimicking medium. The results presented in Figure 5 indicate a significant difference in radiation noise between the EL and the PAA. The SPL of the radiation noise generated by the PAA was substantially lower than that of the EL, owing to the directional nature of the PAA. Additionally, the simplified neck phantom used in this study may have led to some sound leakage at the openings of the trachea. In a more realistic human model, where the trachea is a closed tube, we would expect even lower radiation noise. Moreover, due to the low energy of the difference-frequency sound generated by the PAA, any leaked radiation noise would likely be imperceptible to the listener.

While this study demonstrates the potential of the PAA for reconstructing glottal waveforms and reducing radiation noise, the glottal waveform frequency obtained (200 Hz) remains below the typical fundamental frequency range of normal human speech (60–500 Hz). This limitation is likely due to the output power of the transducer and the modulation method used. Future research should focus on enhancing the output power and improving the conversion efficiency of the difference-frequency signal to achieve a broader frequency range that better matches the natural human voice. Furthermore, the tissue-mimicking phantom in this study does not fully replicate the complex structure of human tissues, particularly regarding their nonlinear acoustic properties. Future studies should explore more anatomically accurate phantoms or in vivo experiments to further validate the feasibility of the parametric acoustic array for practical clinical applications, such as in improving the quality and naturalness of speech in patients with laryngectomy.

The selection of a 5 cm diameter and a 50 kHz center frequency for the transducer in this study was driven by the specific requirements of PAA technology in generating glottal waves within the human body. The diameter was chosen to accommodate the limited area of the human neck while maximizing the energy output of the difference-frequency wave, as the emitting area directly influences the efficiency of PAA energy conversion. Similarly, the 50 kHz center frequency was selected to balance energy conversion efficiency and directivity, with lower frequencies providing higher efficiency but suffering from poor directivity. While the experimental results confirm the feasibility of generating glottal waves within the phantom, further optimization of transducer parameters remains an essential avenue for improving conversion efficiency. Future work will focus on refining transducer size and center frequency, exploring advanced modulation methods, and employing focused transducers to enhance power density at specific target locations, ensuring a stronger and more localized difference-frequency sound field for applications in the human body.

## 5. Conclusions

This study provides compelling evidence that PAA technology is capable of reconstructing glottal waveforms with higher fidelity than traditional EL devices, offering significant advantages in both waveform accuracy and radiation noise reduction. Comparative analysis demonstrated a high degree of similarity between the PAA-generated signals and the model glottal waveforms. Additionally, the autoregressive spectral analysis confirmed that the PAA accurately reproduces essential spectral features of the glottal waveform, further supporting its potential for voice rehabilitation.

The use of a single transducer to generate modulated signals makes this method more efficient and practical for future speech rehabilitation technologies. However, improvements in signal strength, frequency range, and system design are necessary to fully meet the demands of natural human speech. Future work will focus on optimizing transducer parameters, exploring advanced modulation techniques, and conducting clinical evaluations to ensure effective translation of this technology into practical applications. This study lays a strong foundation for advancing voice restoration solutions, with the potential to significantly improve the quality of life for individuals with total laryngectomy.

## Data Availability

Data is unavailable due to privacy or ethical restrictions.
